# Complicated Severe Appendicitis in a 58-Year-Old Female After COVID-19 Infection: A Coincidence or an Association?

**DOI:** 10.7759/cureus.24706

**Published:** 2022-05-03

**Authors:** Zachary Bakhtin, Nikita Agarwal, Luke R Leggett, Francin Alexis, Banu Farabi

**Affiliations:** 1 Internal Medicine, St. Peter's University Hospital, New Brunswick, USA; 2 Internal Medicine, St. Peter’s University Hospital, New Brunswick, USA

**Keywords:** acute abdomen, general surgery, sepsis, covid-19, appendicitis, sars-cov-2, appendectomy

## Abstract

Acute appendicitis (AA) remains the most common cause of acute abdomen worldwide. Although overall mortality in developed countries is low, complication due to perforation, abscess formation, stump appendicitis and intra-abdominal sepsis is associated with increased morbidity. Throughout the COVID-19 pandemic, an increasing proportion of complicated appendicitis has been reported. In this case, we present a 58-year-old female with a remote history of COVID-19 infection and severe appendicitis, complicated by sepsis. Viral infection has previously been proposed as a cause of appendicitis. Our report aims to describe our patient’s course and comment on a potential association with the novel severe acute respiratory syndrome coronavirus 2 (SARS-CoV-2) virus, as well as future diagnostic and management considerations.

## Introduction

Severe acute respiratory syndrome coronavirus 2 (SARS-CoV-2) is a novel beta coronavirus presenting primarily as a respiratory disease; however, it has been documented to have numerous extrapulmonary effects. Increasing reports of gastrointestinal symptoms, along with complications resulting in acute abdomen, have been observed in patients with COVID-19. Cases of acute appendicitis have been seen with increasing rates of perforation, as well as presenting in an atypical fashion [1].

To date, acute appendicitis is still considered the most common cause of surgical abdomen and intra-abdominal sepsis, with up to one-third of cases qualifying as complicated. Severity is typically determined by the presence of perforation, as well as sepsis and peritoneal signs on physical examination [2]. This most commonly results from delay in presentation or diagnosis and can progress to gangrene, intra-abdominal abscess or phlegmon formation [3]. The mean age at diagnosis for appendicitis has increased over time, but the incidence is still generally highest in the second and third decades of life. Despite the decreased incidence of nonperforated appendicitis (coincident with increased CT imaging and laparoscopic appendectomy), there has not been a similar decrease in rates of perforation. This could be related to differing pathophysiological mechanisms between perforated and nonperforated appendicitis [4].

## Case presentation

Our patient is a 58-old-year female with a past medical history of hypertension, hyperlipidemia and COVID-19 infection one month prior, who presented to the emergency department complaining of one-day history of abdominal pain, fever and chills. She described the abdominal pain as a dull sensation, initially over the epigastrium and gradually migrating to the right side of her abdomen. She also reported a fever of 100.3°F at home before coming to the hospital. She stated her last bowel movement was the morning prior and liquid in consistency, however, denied seeing any blood or mucus. She otherwise did not complain of any nausea, vomiting, urinary symptoms, chest pain, shortness of breath or cough.

On initial evaluation, she was noted to be febrile to 103.1°F, tachycardic to 116 beats/min and tachypneic to 30 breaths/min with oxygen saturation of 93% on room air meeting systemic inflammatory response syndrome (SIRS) criteria. Though initially normotensive, blood pressure also trended downward to 92/50 mmHg while in the emergency department. The physical exam was notable for marked tenderness over the right lower quadrant with guarding; the remainder of the exam was unremarkable.

Initial laboratory investigations were notable for transaminitis with aspartate aminotransferase (AST) 168 units/L, alanine aminotransferase (ALT) 86 units/L, alkaline phosphatase 171 units/L and total bilirubin 1.8 mg/dL. No leukocytosis or anemia was noted on complete blood count; however, differential showed 91.0% neutrophils with elevated absolute neutrophil count and bandemia. Coagulation studies showed mildly elevated prothrombin time (PT) of 14.2 seconds and internalized normal ratio (INR) of 1.25, with normal partial thromboplastin time (PTT) of 27.4 seconds. A SARS-CoV-2 RNA polymerase chain reaction (PCR) was also positive; otherwise, the remaining chemistry and lactate were unremarkable.

**Figure 1:**
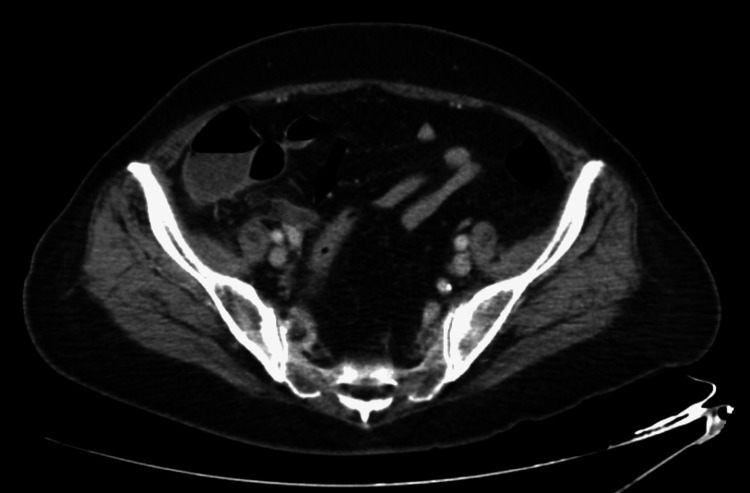
Axial enhanced computed tomography scan showing a distended and fluid-filled distal appendix (black arrow) with wall thickening, as well as surrounding fat stranding.

On imaging, she had a normal portable chest X-ray. Contrast-enhanced CT of the abdomen and pelvis was notable for a fluid-filled and abnormally thickened appendix measuring up to 12 mm with diffuse mural enhancement, as well as mild surrounding fat stranding; incidental findings included a small duodenal diverticulum, small hiatal hernia and uterine fibroids (Figure 1).

The intensive care unit was consulted for sepsis, and general surgery was contacted regarding CT findings demonstrating acute appendicitis. The patient was empirically started on antibiotics and underwent exploratory laparotomy with appendectomy. Infectious disease was consulted and noted that her SARS-CoV-2 RNA PCR could be related to dead virus, rather than active/replicating virus; they recommended following nucleocapsid and spike antibodies. Her postoperative course was otherwise uncomplicated, and blood cultures grew *Streptococcus anginosus* at 30 hours of growth; she was discharged home and completed her course of antibiotics as an outpatient.

## Discussion

As with other inflammatory processes involving hollow organs, inflammation of the wall of the appendix results in increased luminal pressure that causes localized ischemia, which can progress to perforation, abscess formation and peritoneal spread. Obstruction of the appendiceal lumen is the most widely accepted cause leading to inflammation [2]. This can be due to a physical mass, such as a calculus or fecalith, or it can be related to lymphatic hyperplasia, particularly in the setting of infection [5]. In the latter case, reactive lymphoid follicles can cause thickening of the lamina propria, leading to elevated intraluminal pressures. 

Viral infection has long been proposed as a potential cause, with evidence of a temporospatial relationship between infectious outbreaks and appendicitis cases [6]. One retrospective review showed that up to 14% of appendicitis cases had a positive quantitative polymerase chain reaction (qPCR) for adenovirus DNA, while another animal study used immunohistological staining to evaluate for coxsackievirus infection [7-8]. With the rapid spread of SARS-CoV-2 and new variants, more studies comparing appendicitis rates in pandemic epicenters would be helpful. COVID-19 is known to cause both a hypercoagulable state and a dysregulated immune/inflammatory response in severe cases; both are potential mechanisms for causing acute appendicitis (AA). 

Another consideration is the increased proportion of complicated appendicitis seen in SARS-CoV-2-positive patients. Prehospital delays in seeking care and increased requests for nonoperative management have been proposed as explanations for this association but may not fully explain the disparity between uncomplicated and complicated appendicitis [9]. It is possible that this may be due to a direct viral effect and would support the theory that perforated and nonperforated appendicitis could be caused by different pathophysiological mechanisms.

This could also have implications for management, considering the higher frequency of complex AA in COVID-19 patients. Several biomarkers have been found to have a high sensitivity and negative predictive value in diagnosing AA. Early changes in C-reactive protein (CRP) have been shown to have moderate diagnostic value in predicting appendicitis [10]. More recently, ischemia-modified albumin (IMA) has been used in the diagnosis of early myocardial ischemia and other ischemic diseases. Kılıç et al. also noted a strong positive correlation between IMA levels and gangrenous/perforated appendicitis [11]. Obtaining IMA levels in SARS-CoV-2-positive patients could be helpful in predicting disease severity, particularly in cases where access to CT imaging is more limited.

## Conclusions

This report presented a case of severe appendicitis in an older patient with a remote history of COVID-19 infection and a persistently positive SARS-CoV-2 PCR. We have seen a rise in the proportion of complicated appendicitis cases during the pandemic, which may only be partially explained by delays in seeking care. At this point, it is unclear if the SARS-CoV-2 virus has a direct effect on the development of AA or otherwise predisposes the patient to it. Further study is needed to determine what involvement, if any, SARS-CoV-2 may have in the pathophysiology of appendicitis.
